# Chemotherapy for Hodgkin's lymphoma in a patient receiving clozapine for treatment-resistant schizophrenia: use of the Mental Capacity Act 2005

**DOI:** 10.1192/pb.bp.114.048306

**Published:** 2015-12

**Authors:** Florence Elizabeth Chamberlain, Nicholas Walsh, Jan Falkowski

**Affiliations:** 1 North East Thames Foundation School, UK; 2 Newham General Hospital, London, UK; 3 Mile End Hospital, London, UK

## Abstract

Treatment resistance occurs in approximately 30% of individuals with schizophrenia and is commonly treated with clozapine. Nodular sclerosing Hodgkin's lymphoma is a subtype of Hodgkin's lymphoma predominantly affecting those under 50 years of age. In this case report, an individual with treatment-resistant schizophrenia developed nodular sclerosing Hodgkin's lymphoma and is treated with concurrent clozapine and systemic chemotherapy. The aim of this case report is to act as guidance for clinicians and to outline the difficulties of treating individuals with psychiatric illness under the Mental Capacity Act 2005 when the proposed treatment could lead to high levels of morbidity and mortality.

Nodular sclerosing Hodgkin's lymphoma is the commonest subtype of Hodgkin's lymphoma.^1^ It is characterised by proliferation of malignant lymphocytes in the reticuloendothelial system and presenting with lymphadenopathy or systemic ‘B’ symptoms.^2^ It affects men and women equally and predominantly those under 50 years.^1^ There are 3 cases of Hodgkin's lymphoma per 100 000 people in England,^3^ and without treatment 5-year survival ranges from 40 to 95%.^2,4^ Treatment includes radiotherapy, systemic chemotherapy or both^2^ and is curative in 90% of cases.^1^ Risks from systemic chemotherapy treatment include myelosuppression, febrile neutropaenia and thrombocytopaenia.^2^ The mortality rate from febrile neutropaenia is 9.5%.^5^

The Mental Capacity Act 2005 is a legal framework in England and Wales for making decisions on behalf of adults who lack capacity to make particular decisions for themselves. All adults are presumed to have capacity for a particular decision unless it can be proven otherwise. Patients who lack capacity require their nearest relative or an independent mental capacity advocate (IMCA) to make a decision on their behalf. Under certain circumstances, decisions are made by the Court of Protection. Treatment under the Mental Health Act 1983 does not include the treatment of physical illness except in cases where the mental illness is a consequence of a physical illness.

Treatment resistance is found in approximately 30% of patients with schizophrenia.^6^ Clozapine is currently the most effective management option in schizophrenia, but because of its side-effect profile it is reserved for use only as a third-line agent.^6-9^ This includes a 0.3-2% risk of potentially fatal agranulocytosis,^8-10^ which is higher among the Asian population^8,11^ and carries a mortality rate of 5-10%.^12^ Despite this, a delay in starting clozapine in those with treatment-resistant schizophrenia has been shown to lead to poor outcomes.^9,13^

To reduce the risks associated with the development of agranulocytosis, blood parameters should be monitored closely^14^ and clozapine should be discontinued permanently if leukocyte counts drop significantly.^10^ During clozapine treatment clinicians should avoid prescribing drugs that depress leucopoiesis.^10^ Here we describe an unusual case where a patient was successfully treated for nodular sclerosing Hodgkin's lymphoma using the Mental Capacity Act 2005 while concurrently treated with clozapine under Section 3 of the Mental Health Act.

## Case presentation

A Bangladeshi patient with a 20-year history of treatment-resistant schizophrenia was treated with clozapine. Since diagnosis, the patient has had frequent admissions to acute psychiatric hospitals due to non-adherence, leading to rapid relapse of psychotic symptoms within days of cessation. However, when adherent to clozapine, the patient remains relatively independent with only mild residual delusional ideas persisting in the background.

An abnormal swelling developed in the patient's neck, but believing this to be a side-effect of clozapine, the patient refused to comply with further treatment. While admitted as an in-patient under Section 3 of the Mental Health Act 1983, the patient was diagnosed with stage IIA nodular sclerosing Hodgkin's lymphoma and managed expectantly. However, the patient developed severe leg oedema secondary to inferior vena cava syndrome. Meanwhile, the lymphoma progressed to stage IIIB, with a very poor prognosis if untreated.

The patient refuted the diagnosis of Hodgkin's lymphoma and accounted for symptoms using a multitude of delusional ideas. These included being ‘incriminated by someone – who tried to get into my blood and body’ and saying that their neck was ‘just bloated’. It was deemed that the patient did not have the capacity to consent to or refuse treatment for Hodgkin's lymphoma at that time. The patient was unable to make use of the information that without treatment, a likely outcome would be death, but with treatment the prognosis was excellent. They were not able to understand the gravity of their illness, or why treatment was being offered, as well as continuing to deny the diagnosis. The patient could not weigh up the information provided but was, however, adamant that they did not want their life to end.

The risk to the patient and to others from a prolonged schizophrenia relapse if clozapine was withheld during chemotherapy treatment was weighed against risk of death from myelodepletion. Switching to an alternative antipsychotic agent was considered but not possible due to failure of multiple first- and second-line agents in the past. A literature search failed to provide any evidence of agranulocytosis from treating patients with clozapine and systemic chemotherapy previously.^15^ It was therefore deemed in the patient's best interests to give systemic chemotherapy and clozapine concurrently.

The urgency of treating the Hodgkin's lymphoma took precedence over waiting to see whether capacity could be regained with sustained clozapine adherence. In the absence of a nearest relative to consult, an IMCA was appointed, who agreed that systemic chemotherapy treatment should progress immediately against the patient's wishes in their best interests. Application was successfully made to treat the patient with clozapine off-licence.

Before commencing systemic chemotherapy, haematological parameters and clozapine level were within normal range. The patient was treated with six cycles of systemic chemotherapy. Each cycle consisted of doxorubicin 25 mg/m^2^, bleomycin 10 000 iu/m^2^, vinblastine 6 mg/m^2^ and dacarbazine 375 mg/m^2^ on day 1 and day 15 with a 28-day break between cycles. During the first cycle of systemic chemotherapy the patient became agitated and required sedation. It was decided that continuing with further treatment should be decided by the Court of Protection, which granted permission.

The patient completed treatment successfully and obtained complete remission from Hodgkin's lymphoma 6 months after treatment completion.

## Discussion

This case illustrates some of the practical difficulties in treating patients under the Mental Capacity Act 2005. Although the probable outcome of not treating this patient's lymphoma was death, the proposed treatment also carried a high risk of morbidity and mortality and the administration of cytotoxic agents requires total compliance on behalf of the patient to avoid causing unnecessary harm to the patient and staff administering the treatment.

Any proposed intervention should be the least restrictive of the patient's basic rights and freedoms and the patient must be given all appropriate help to restore capacity to make such a decision. In this case, there was no time to spare to see whether reinitiating clozapine would restore the patient's capacity to consent to treatment and avoid application to the Court of Protection to treat without the patient's consent.

Since a third of the general population will be diagnosed with cancer in their lifetime^16^ and the population prevalence of schizophrenia is 0.30-0.66%,^17^ the concurrent use of clozapine and systemic chemotherapy in Hodgkin's lymphoma and other malignancies needs formal investigation. Our current knowledge base is from a handful of case reports which are not necessarily relevant to the malignancy in question.^15,18^ There is, however, a growing body of evidence from case reports that it may be safe to reintroduce clozapine treatment to control psychotic symptoms once the patient is established on systemic chemotherapy treatment.^19-21^ One case report exists where clozapine was reinstated in a case of Hodgkin's lymphoma when other antipsychotic agents failed to maintain psychotic symptom remission.^22^ But there is a lack of clear national and local guidelines as to the safe monitoring of these two potentially lethal treatments when prescribed concurrently. In the case of the patient we have described, blood parameters and clozapine levels were measured twice weekly and physical observations conducted 4-hourly throughout treatment.

It is widely established that patients are at greatest risk of agranulocytosis in the first 6 months of treatment with clozapine. A few cases of delayed-onset agranulocytosis have been described.^23,24^ The mechanism remains unknown, and thus physicians and psychiatrists are reluctant to prescribe concurrent myelosuppressing medication.^21^ Whether those well established on clozapine therapy are less likely to develop agranulocytosis while receiving systemic chemotherapy than those within 6 months of treatment needs also to be investigated.

Lithium has been used to increase neutrophil counts in those with neutropaenia during systemic chemotherapy and also in those with neutropaenia from clozapine therapy.^8,9,25^ The mechanism is poorly understood and may lead to an increased risk of lithium toxicity despite levels being within the therapeutic range.^8^ There is also some evidence that granulocyte-colony stimulating factor (G-CSF) can be used to treat agranulocytosis associated with chemotherapy and clozapine.^8^ However, whether lithium and G-CSF can be used prophylactically in cases where systemic chemotherapy and clozapine are concurrently prescribed has not been examined.

## Conclusion

Despite the fact that none of the haematological parameters fell below critical levels in our patient, further research is needed before full conclusions with regard to safety during concurrent clozapine and systemic chemotherapy treatment. This case demonstrates the importance of regular immune and haematological parameters monitoring when such treatments are proposed.
